# Typing Method for the QUB11a Locus of* Mycobacterium tuberculosis*: IS*6110* Insertions and Tandem Repeat Analysis

**DOI:** 10.1155/2016/5216530

**Published:** 2016-10-16

**Authors:** Eriko Maeda-Mitani, Koichi Murakami, Akira Oishi, Yoshiki Etoh, Nobuyuki Sera, Shuji Fujimoto

**Affiliations:** ^1^Fukuoka Institute of Health and Environmental Sciences, 39 Mukaizano, Dazaifu, Fukuoka 818-0135, Japan; ^2^Infectious Disease Surveillance Center, National Institute of Infectious Diseases, 4-7-1 Gakuen, Musashimurayama, Tokyo 208-0011, Japan; ^3^Department of Health Sciences, Faculty of Medical Sciences, Kyushu University, 3-1-1 Maidashi, Higashi-ku, Fukuoka 812-8582, Japan

## Abstract

QUB11a is used as a locus for variable number of tandem repeats (VNTR) analysis of* Mycobacterium tuberculosis* Beijing lineage. However, amplification of QUB11a occasionally produces large fragments (>1,400 bp) that are not easily measured by capillary electrophoresis because of a lack of the typical stutter peak patterns that are used for counting repeat numbers. IS*6110* insertion may complicate VNTR analysis of large QUB11a fragments in* M. tuberculosis*. We established a method for determining both tandem repeat numbers and IS*6110* insertion in the QUB11a locus of* M. tuberculosis* using capillary electrophoresis analysis and* Bsm*BI digestion. All 29 large QUB11a fragments (>1,200 bp) investigated contained IS*6110* insertions and varied in the number of repeats (18 patterns) and location of IS*6110* insertions. This method allows VNTR analysis with high discrimination.

## 1. Introduction

QUB11a, or variable number of tandem repeats (VNTR) locus 2163a, is an analytical locus that has been useful for molecular epidemiological analysis of the Beijing lineage of* Mycobacterium tuberculosis *[1, 2]. Beijing lineage, which is a homogenous strain group [2], is the dominant lineage in Asia [3, 4] and Russia [5] and the most prevalent genotype worldwide [6]. Allix-Béguec et al. found that amplification of QUB11a occasionally produced large fragments (>1,400 bp) [7] that were not easily measured by capillary electrophoresis because of a lack of the typical stutter peak patterns, although they also demonstrated the high discriminatory power of QUB11a [7]. Despite this, QUB11a was not included in the international typing scheme based on 24 VNTR loci used for* M. tuberculosis* [8]. However, Wada et al. [9] and Millet et al. [1] also showed the utility of QUB11a for discrimination of Beijing lineage in Japan, and Vlji et al. recommended the use of hypervariable loci, including QUB11a, for VNTR analysis of Beijing lineage in the United Kingdom [2]. Based on these findings, QUB11a is not an inconsequential locus.

IS*6110* (approximately 1,360 bp) [10] is found in the QUB11a locus of* Mycobacterium caprae* [11], leading us to hypothesize that it may also be present in the corresponding region of* M. tuberculosis*. Insertion would likely complicate VNTR analysis of large QUB11a fragments in* M. tuberculosis*, indicating that a method for analyzing these large QUB11a fragments may increase the discriminatory power of* M. tuberculosis* typing by VNTR. Therefore, the aims of the present study were to determine the presence or absence, frequency, location, and stability of IS*6110* insertions in the QUB11a locus of* M. tuberculosis* isolates and to develop a VNTR typing method for the QUB11a locus.

## 2. Materials and Methods

### 2.1. Isolates and Analysis of the QUB11a Locus

VNTR analysis of QUB11a from 312* M. tuberculosis* isolates collected from humans in Fukuoka Prefecture (*n* = 306) and neighboring prefectures (*n* = 6), Japan, between 2012 and 2013 was performed as described previously [12, 13]. The isolates were obtained from 312 newly diagnosed patients aged from 17 to 102 years (average: 68.3 years; median: 76 years) from eight separate hospitals (127 females and 185 males). In the same period, 944 patients were reported as newly diagnosed in Fukuoka Prefecture as a whole.

**Figure 1:**
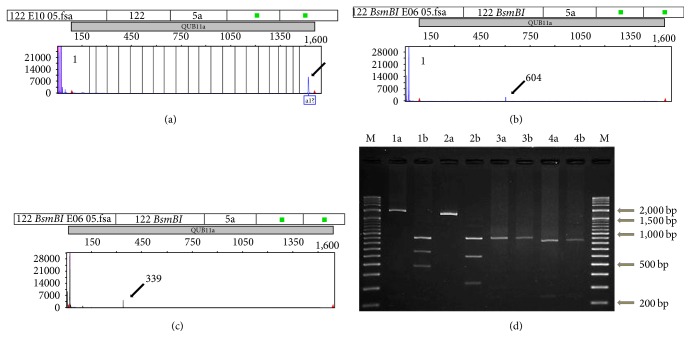
Electrophoresis of a* Bsm*BI-digested large QUB11a fragment amplified from a single* Mycobacterium tuberculosis* isolate using primers labeled with fluorescent dyes FAM and NED. (a–c) Capillary electrophoresis. (d) Agarose gel electrophoresis. (a) The untreated QUB11a region fragment is outside the limits of the size marker (>1,200 bp) of GeneMapper software (Life Technologies, Carlsbad, CA, USA). Therefore, size calculations generated by the software are unreliable. (b and c) Fragments generated following digestion of QUB11a amplicons with* Bsm*BI. Two fragments, labeled with fluorescent dyes FAM (b) and NED (c), were detected. (d) Agarose gel (2%) electrophoresis analysis of QUB11a loci from four isolates. Lanes 1a and 2a: untreated QUB11a amplicons (>1,200 bp); lanes 1b and 2b: QUB11a amplicons treated with* Bsm*BI; lanes 3a and 4a: untreated QUB11a amplicons (<1,200 bp); lanes 3b and 4b: QUB11a amplicons treated with* Bsm*BI. Lane M, mixed 100 bp and 500 bp DNA ladders.

Amplification products corresponding to the QUB11a locus were analyzed by capillary electrophoresis (3500 Genetic Analyzer, Life Technologies, Carlsbad, CA, USA) and GeneMapper software (Life Technologies) to determine the fragment sizes (the combination of electrophoresis and measurement is hereafter referred to as capillary electrophoresis analysis). GeneScan 1200 LIZ Size Standard (Life Technologies) was used as an internal size marker. Analysis showed that 29 (9.3%) of the isolates produced large fragments (>1,200 bp) (Figure 1(a)). These large fragments corresponded to a peak located outside of the internal size marker range of GeneMapper software (Figure 1(a)) and therefore could not be measured. These 29 isolates were further analyzed in the following assays.

### 2.2. IS*6110* Detection in QUB11a Using Sanger Analysis

VNTR numbers and presence or absence and location of the IS*6110* insertion in QUB11a from the 29 isolates were determined by Sanger analysis (3500 Genetic Analyzer, Life Technologies). The large fragment was amplified from all 29 isolates using primers iwamoto-F and iwamoto-R as described previously [13] and then Sanger-sequenced using the primers iwamoto-F, iwamoto-R [13], INS1, INS2 [14], Nandagopal-F, Nandagopal-R [15], ISseq-F (TACTACGCTCAACGCCAGAG), and ISseq-R (TACCTCCTCGATGAACCACC) and a BigDye Terminator v3.1 Cycle Sequencing Kit (Life Technologies).

### 2.3. Stability Test of IS*6110*

To confirm the stability of IS*6110* in the QUB11a locus, 26 of the 29 isolates were passaged in 2% Ogawa's medium (Kyokuto Pharmaceutical Industrial Co., Tokyo, Japan) every 2 weeks for 20 weeks to give a total of 10 passages (stability test).

### 2.4. IS*6110* Detection in QUB11a Using a Simplified Method

**Figure 2:**
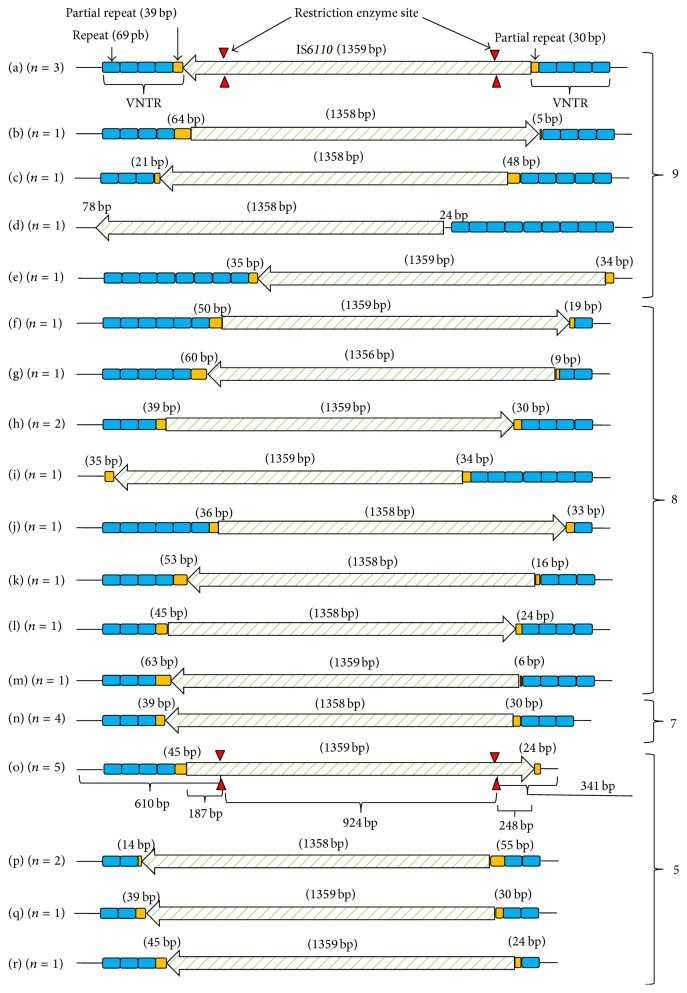
Schematic representation of QUB11a loci with IS*6110* insertions in* Mycobacterium tuberculosis*. Sequence patterns for the 29 tested* M. tuberculosis* isolates were classified into 18 groups. Thin black line, square, and arrow indicate flanking sequence, tandem repeats, and IS*6110*, respectively. Triangles indicate* Bsm*BI restriction enzyme recognition site in IS*6110*. Numbers located on the right side of the schematic indicate tandem repeat number of each sequence pattern.

We also aimed to establish a simple method for detection of the IS*6110* insertion, along with tandem repeat number(s), in the QUB11a locus based on capillary electrophoresis analysis of* Bsm*BI-digested fragments (Figure 2(o)).* Bsm*BI was predicted to digest the IS*6110* region into three segments (approximately 925 bp, 250 bp, and 190 bp) but not cut the QUB11a repeat region. The QUB11a locus was amplified from genomic DNA from the 29 isolates using primers iwamoto-F (forward) and iwamoto-R (reverse), which were labeled with fluorescent dyes fluorescein (FAM) and NED, respectively. The amplified fragments were treated with* Bsm*BI (New England Biolabs, Ipswich, MA, USA) at 37°C for 1 h. The* Bsm*BI-treated fragments were then analyzed simultaneously by capillary electrophoresis, as described above.

### 2.5. Ethics Statement

This study was carried out in strict accordance with the guidelines of the Ethics Regulations Related to Epidemiological Research at the Fukuoka Institute of Health and Environmental Sciences, which is based on domestic standards (the Ethical Guidelines for Epidemiological Research, 17 June 2002, Ministry of Education, Culture, Sports, Science and Technology and Ministry of Health, Labour, and Welfare, Japan), and approved by the Ethics Committee of Fukuoka Institute of Health and Environmental Sciences under permit number 26-9. The strains tested were anonymized and donated by the hospitals.

## 3. Results

### 3.1. IS*6110* Detection in QUB11a Using Sanger Analysis

All large QUB11a fragments (29/29) contained IS*6110 *insertions and varied in the number of repeats (18 patterns) and the locations of IS*6110 *insertions (Figure 2). IS*6110* was inserted within the repeat region of QUB11a in all isolates except one (Figure 2(d)), in which IS*6110* was localized to a region flanking the tandem repeats.

### 3.2. Stability of IS*6110*

The number of repeats and the presence and location of IS*6110* in QUB11a did not change in any of the 26 isolates during the 10 passages.

### 3.3. IS*6110* Detection in QUB11a Using the Simplified Method

When the large fragment of QUB11a (Figure 1(a)) was digested with* Bsm*BI, two of the expected fragments were detected by capillary electrophoresis (Figures 1(b) and 1(c)), and agarose gel electrophoresis (2% agarose) confirmed the presence of the third fragment (Figure 1(d)). Subsequently, each amplicon produced two labeled fragments and an intervening unlabeled fragment (Figure 2(o)). Therefore, based on the size of the fragments detected by capillary electrophoresis analysis, the number of tandem repeats in the region can be calculated, in addition to determining the presence of the IS*6110* insertion. For example, fragments (a)–(c) shown in Figure 1 correspond to fragment (o) in Figure 2, where the detected product in Figure 1(b) (approximately 604 bp) and the product in Figure 1(c) (approximately 339 bp) correlate with the 610 bp and 341 bp products, respectively, of the digestion of fragment (o) (Figure 2) with* Bsm*BI. Therefore, the size of the PCR product is calculated as follows: 604 + 339 + 925 = 1,868 (bp), or detected fragment length with labeled forward PCR primer (bp) + detected fragment length with labeled reverse PCR primer (bp) + theoretical length of IS*6110* portion (bp) = estimated length of the amplified QUB11a locus (bp). Next, the number of repeats was determined as follows: (1,868 − (1,359 + 171))/69 = 4.898. In the calculation, the figures used were obtained as follows: 1,868: detected length (bp) of the amplified QUB11a locus; 1,359: length of IS*6110 *(bp); 171: length of flanking sequence (bp); and 69: repeat unit length (bp) of tandem repeats in the QUB11a fragment (bp). The capillary electrophoresis method used detects repeats (including the IS*6110 *region) as well as contiguous sequences (flanking sequences, 171 bp) in the QUB11a locus.

### 3.4. Comparison of the Two Analysis Methods

**Figure 3:**
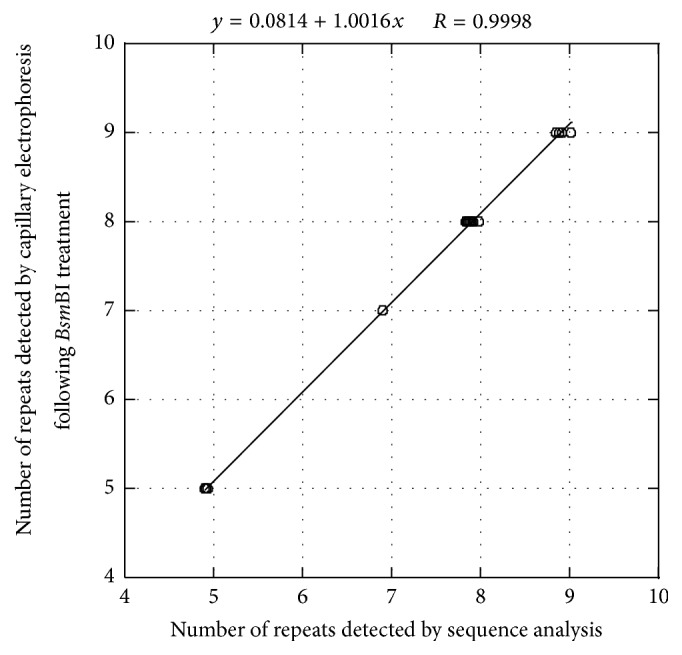
Comparison of the tandem repeat numbers, excluding IS*6110*, obtained from capillary electrophoresis of* Bsm*BI-digested QUB11a amplicons with those from Sanger analysis of QUB11a loci from the 29 isolates. A simple linear regression was obtained, where *x* is the number of tandem repeats estimated by Sanger analysis and *y* is the number estimated by capillary electrophoresis. Graphing and data analysis was carried out using KaleidaGraph (Synergy Software, Reading, PA, USA).

The repeat numbers estimated by the capillary electrophoresis analysis method were then compared with those estimated by Sanger analysis for all 29 isolates (Figure 2). Results showed that there were some discrepancies between the two methods (Figure 3). Regression analysis was then carried out on the results obtained using the two different methods. A simple linear regression formula was obtained using KaleidaGraph (Synergy Software, Reading, PA, USA): *y* = 0.0814 + 1.0016*x*, *R* = 0.9998 (where *y* and *x* are the numbers of tandem repeats calculated from capillary electrophoresis results and Sanger analysis results, resp.). Using this regression equation, the isolate that produced fragments (a)–(c) in Figure 1 was estimated to contain 4.99 repeats, which agreed with the results of DNA sequencing of fragment (o) shown in Figure 2 (five repeats). Reproducibility of the experiment was confirmed by triplicate capillary electrophoresis analysis of* Bsm*BI-digested PCR products from eight amplified QUB11a loci. Incidentally, the amplified QUB11a fragments from four isolates were much smaller (<1,200 bp) than the rest of the isolates and were not cleaved by* Bsm*BI (3a, 3b, 4a, and 4b in Figure 1(d)). These novel QUB11a sequences were deposited in the DNA Data Bank of Japan under accession numbers LC005454–LC005482.

## 4. Discussion

In the present study, we established a method to detect IS*6110* and count the number of tandem repeats in large QUB11a fragments in* M. tuberculosis*. Information regarding the presence or absence of an IS*6110* insertion in the locus will provide an additional way to distinguish between isolates, along with the number of repeats. This new method takes less than 7 h (including amplification of template DNA, digestion of PCR product by* Bsm*BI, separation by capillary electrophoresis, and calculation of the number of tandem repeats) and increases the accuracy and discriminatory power of VNTR typing at the QUB11a locus of* M. tuberculosis*.

Although IS*6110* sequences vary [16], we also carried out* in silico* analysis of 229 elements from 17* M. tuberculosis* strains available from GenBank (https://www.ncbi.nlm.nih.gov/nucleotide, accessed on 10 October 2014; accession numbers AL123456, CP002871, CP002882–CP002885, CP003233, CP003234, CP004886, CP005082, CP005386, CP007809, NC_002755, NC_009525, NC_020089, NC_020559, and NC_021740) and showed that all (229/229) of these regions contained the two* Bsm*BI restriction sites (data not shown). Moreover, the present study showed that IS*6110* in the QUB11a locus is relatively stable, which is consistent with a previous report [10]. Therefore, the method described here may be applicable to most* M. tuberculosis* isolates.

Discrimination between strains of Beijing lineage is important for countries and areas where Beijing lineage is dominant [1, 2]. Using our method, two isolates in group A exhibited the same VNTR pattern when 24 loci were examined: Mtub4, MIRU10, Mtub21, Mtub24, QUB11b, VNTR2372, MIRU26, QUB15, MIRU31, QUB3336, QUB26, QUB4156, ETR A, QUB18, QUB3232, VNTR3820, VNTR4120, MIRU04, MIRU16, MIRU40, ETR C, Mtub30, Mtub39, and QUB11a (nine repeats + IS*6110*) (data not shown). The present method typed QUB11a regions correctly, and the 24-locus typing assay revealed the possibility of epidemiological linkage of these isolates. Although our method might not be applicable for all other strains that harbor large QUB11a fragments, it may contribute to expanding the discriminatory power of VNTR for* M. tuberculosis* Beijing lineage.
